# The Human Paraoxonase 2: An Optimized Procedure for Refolding and Stabilization Facilitates Enzyme Analyses and a Proteomics Approach

**DOI:** 10.3390/molecules29112434

**Published:** 2024-05-22

**Authors:** Eros A. Lampitella, Maria Marone, Nagendra S. K. Achanta, Elena Porzio, Francesco Trepiccione, Giuseppe Manco

**Affiliations:** 1 Institute of Biochemistry and Cell Biology-CNR, Via Pietro Castellino 111, 80131 Naples, Italy; erosantonio.lampitella@ibbc.cnr.it (E.A.L.); maria.marone@ibbc.cnr.it (M.M.); nagendrasaiachanta@gmail.com (N.S.K.A.); elena.porzio@cnr.it (E.P.); 2 Department of Translational Medical Science, University of Campania “Luigi Vanvitelli”, Via Leonardo Bianchi c/o Ospedale Monaldi, 80131 Naples, Italy; francesco.trepiccione@unicampania.it

**Keywords:** paraoxonase, lactonase, enzyme kinetics, biofilm, quorum quenching

## Abstract

The human paraoxonase 2 (PON2) is the oldest member of a small family of arylesterase and lactonase enzymes, representing the first line of defense against bacterial infections and having a major role in ROS-associated diseases such as cancer, cardiovascular diseases, neurodegeneration, and diabetes. Specific Post-Translational Modifications (PTMs) clustering nearby two residues corresponding to *pon2* polymorphic sites and their impact on the catalytic activity are not yet fully understood. Thus, the goal of the present study was to develop an improved PON2 purification protocol to obtain a higher amount of protein suitable for in-depth biochemical studies and biotechnological applications. To this end, we also tested several compounds to stabilize the active monomeric form of the enzyme. Storing the enzyme at 4 °C with 30 mM Threalose had the best impact on the activity, which was preserved for at least 30 days. The catalytic parameters against the substrate 3-Oxo-dodecanoyl-Homoserine Lactone (3oxoC12-HSL) and the enzyme ability to interfere with the biofilm formation of *Pseudomonas aeruginosa* (*PAO1*) were determined, showing that the obtained enzyme is well suited for downstream applications. Finally, we used the purified rPON2 to detect, by the direct molecular fishing (DMF) method, new putative PON2 interactors from soluble extracts of HeLa cells.

## 1. Introduction

The human paraoxonase (PON) family encompasses three members, i.e., PON1, PON2, and PON3, which are encoded by three different genes located in a cluster on chromosome 7 [1] and are highly conserved within and between species [2]. Phylogenetic analysis showed that PON2 is the oldest member of the family and that PON1 and PON3 have evolved from it [3]. Paraoxonases have different functions and are found at different intracellular locations [4]. PON1 and PON3 are primarily expressed in the liver and associated with high-density lipoprotein (HDL) in the blood stream. PON2, instead, is ubiquitously expressed [4,5] and associated with the plasma membrane, where it counteracts lipid peroxidation [6], as well as other intracellular membranes, in the nucleus, endoplasmic reticulum (ER), and mitochondria [7]. Despite the name “paraoxonase”, only PON1 has a good paraoxonase activity [8]. However, all three PONs mainly exhibit lactonase activity, collateral arylesterase activity, and a redox function, which reduces the levels of reactive oxygen species (ROS) [7]. PON2 prevents the ubisemiquinone-mediated mitochondrial superoxide generation in the mitochondria and consequent apoptosis, independent of its lactonase activity [9].

At the mRNA level, seven isoforms have been described [1,10], but only three seem to be present at the protein level, at least in Hela cells [11]: the canonical full-length protein; the isoform 1 with different residues in the sequence 1–16 [1]; and a third isoform, missing sequence residues 123–134, which by a SAXS analysis appears to be unstructured and mostly inactive in vitro [11,12]. At least two *pon2* single-nucleotide polymorphisms (SNPs), corresponding to A148G and S311C, have been reported to be associated with decreased lactonase activity and alterations in plasma lipid levels in patients affected by human diseases related to oxidative stress such as coronary artery disease (CAD) [13,14,15], type 2 diabetes mellitus, Alzheimer’s disease [16,17], cataracts [18], and COVID-19 [19]. PTMs, mainly ubiquitinations, were found to gather nearby 148 and 311 residues and are probably involved in modulating the PON2 activity [13]. Ubiquitination at position 144 has been shown to be responsible for the 3oxoC12-HSL-mediated PON2 partial inactivation [18,20]. A model for the control of PON2 expression via a putative mRNA operon involving the Wilms’ Tumour 1-Associating Protein (WTAP) and the E3-ubiquitin ligase baculoviral IAP repeat-containing (BIRC3) protein has been recently described [11], offering a link to several different pathways such as AP-1/JNK and PI3K/PDGFR-β signaling. PON2 seems to represent the first line of defense against pathogens, attenuating infection through 3oxoC12-HSL hydrolysis, thus it is considered a part of the cell innate immunity [18]. The 3oxoC12-HSL is of fundamental importance for bacterial infections, representing a key signal and acting as the master regulator of quorum sensing (QS) gene control. This signal is necessary for the biofilm maturation and the full expression of virulence factors, including pyocyanin and elastase [21,22]. Inside the biofilm, several mechanisms confer to bacteria multi-factorial resistance to antibiotics [23]. The inhibition of QS signal using chemical or enzymatic means, a strategy known as quorum quenching (QQ), is the preferred way to combat microbial infections since it attenuates the pathogenicity of microbes and enhances the microbial biofilm susceptibility to antibiotics [24], without eliciting further drug resistance [25]. However, some bacteria are thought to be able to exploit cellular defense mechanisms to protect themselves, using 3oxoC12-HSL to acidify the cytosol and to trigger Ca^2+^ release, which in turn reduces reversibly the PON2 hydrolytic activity [26]. A fair amount of purified protein is required for more in-depth biochemical and structural studies that are needed to link together the PTMs, the modulation of catalytic activity, and the role of SNPs. Moreover, PON2 represents a potential biotechnological enzyme for the treatment of biofilm-sustained bacterial infections and as such strategies for large-scale enzyme production are necessary. Thus, the main goal of the present study was improving the previously reported purification procedure of a recombinant form of PON2 (rPON2) in *E. coli* [20], as well as testing several chemical additives to ameliorate the poor stability of the protein in solution. Consequently, we could further characterize the purified protein in its ability to degrade 3oxoC12-HSL and to inhibit *P. aeruginosa* biofilm formation in vitro, showing that the enzyme presents characteristics that make it suitable for a potential biotechnological application. Finally, the availability of the recombinant protein allowed mass spectrometry to distinguish PON2 interactors involved in several key cellular pathways from a soluble extract of HeLa cells.

## 2. Results

### 2.1. rPON2 Refolding and Purification

**Figure 1:**
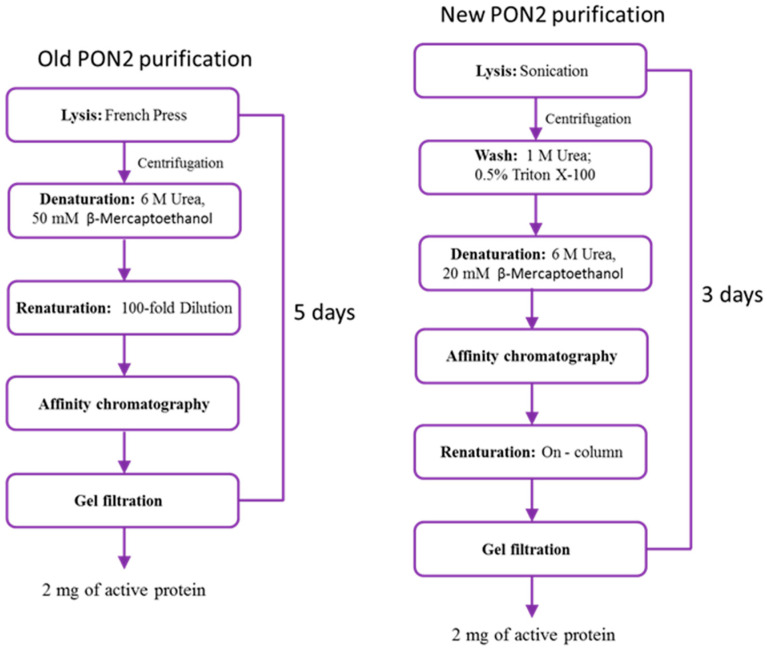
Comparison between the previously reported purification procedure of rPON2 according to Mandrich et al. [20] (on the (**left side**)) and the new purification procedure reported in this work (on the (**right side**)).

We improved the protocol reported by Mandrich et al. [20] for the extraction and refolding of rPON2 from *E. coli* inclusion bodies. A comparison between the two procedures is reported in Figure 1.

The major differences introduced are listed below:

1. Cells were lysed by low-amplitude sonication to preserve the inclusion bodies, in the substitution of French press cell disruption.
2. A series of washes with low concentrations of Triton X-100 and urea were added to remove contaminants bound to inclusion bodies through non-specific interactions [27].
3. The protein–resin binding step was performed with the protein in its unfolded state, in which the his-tag is exposed, to decrease the time of the binding compared to previous conditions.
4. Column renaturation was chosen for protein refolding in the substitution of the 100-fold dilution method used before, to overcome the problem of handling huge volumes of sample.

The purification method reported in this work is quicker, with similar yields, and hence more suitable for future studies.

**Figure 2:**
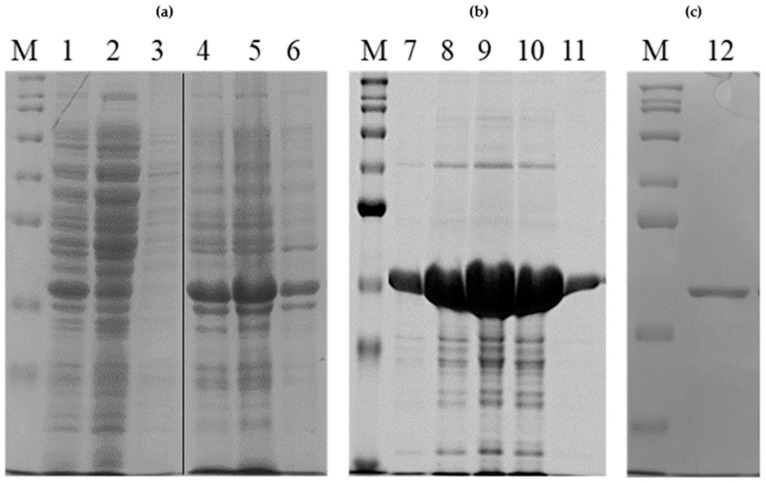
**rPON2 purification analysis by SDS-PAGE.** Panel (**a**), lane 1: total IPTG-induced cell lysate. Lane 2: supernatant after centrifugation. Lane 3: Triton-X100 wash of pelleted inclusion bodies. Lane 4: total inclusion bodies. Lane 5: solubilized inclusion bodies. Lane 6: non-solubilized inclusion bodies. Panel (**b**), lane M: molecular weight markers. Lanes 7–11: fractions eluted from Ni-NTA column. Panel (**c**), lane M: molecular weight markers. Lane 12: purified protein after gel filtration (see Figure 3).

When expressed in the bacterial host, rPON2 is present almost exclusively in an insoluble form, within the inclusion bodies [20]. Treatment of the isolated inclusion bodies with high concentrations of urea yield a soluble, albeit denatured, rPON2. A Sodium Dodecyl Sulfate–Polyacrylamide Gel Electrophoresis (SDS-PAGE) analysis (Figure 2) showed the presence of a band corresponding to the molecular weight of rPON2 (~37 kDa), which is consistent with the absence of PTMs.

After a single nickel–nitriloacetic acid (NTA) affinity purification step, followed by column renaturation (see Section 4 for details), the protein presented a specific activity of 0.27 ± 0.25 U/mg for 3oxoC12-HSL.

**Figure 3:**
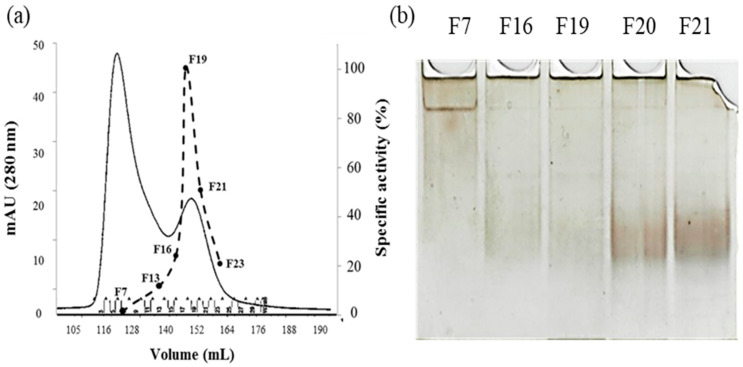
(**a**) **Size-exclusion chromatography purification step of rPON2 on 26/600 Superdex G-75 column**. Protein was assessed by absorbance reading at 280 nm (continuous trace) and by activity measurements with the substrate 3oxoC12-HSL (dotted trace); (**b**) **Native PAGE** highlighting the presence of a multimer–monomer equilibrium. F7 represents the fraction corresponding to the first peak (Peak 1) of size-exclusion chromatography, while F16 to F21 are the fractions corresponding to the second peak (Peak 2).

Gel chromatography (Figure 3a) and Native PAGE (Figure 3b) highlighted the presence of a dimer–monomer equilibrium.

Peak 1 of gel filtration chromatogram, eluting at a volume corresponding to a relative mass of 75 kDa, contained rPON2 as well as other contaminants, suggesting that the protein is in a dimeric form. The specific activity of this peak’s fractions on 3oxoC12-HSL was 0.060 ± 0.015 U/mg. Peak 2 instead eluted at a volume corresponding to a mass of 37–40 kDa and constitutes exclusively of a single band (Figure 2), indicating that the protein is present in the monomeric form. The specific activity for 3oxoC12-HSL of this fraction was 0.55 ± 0.08 U/mg, which was comparable to the full-length protein expressed in insect cells by Draganov et al. 2005 [8]. A Native PAGE analysis (Figure 3b) showed that, in the fractions from Peak 1, there was a predominant band at a higher molecular weight, probably corresponding to a higher order multimer, while in the fractions from the second peak, there was a diffuse band at a lower molecular weight, probably corresponding to different conformations of the protein monomer.

**Figure 4:**
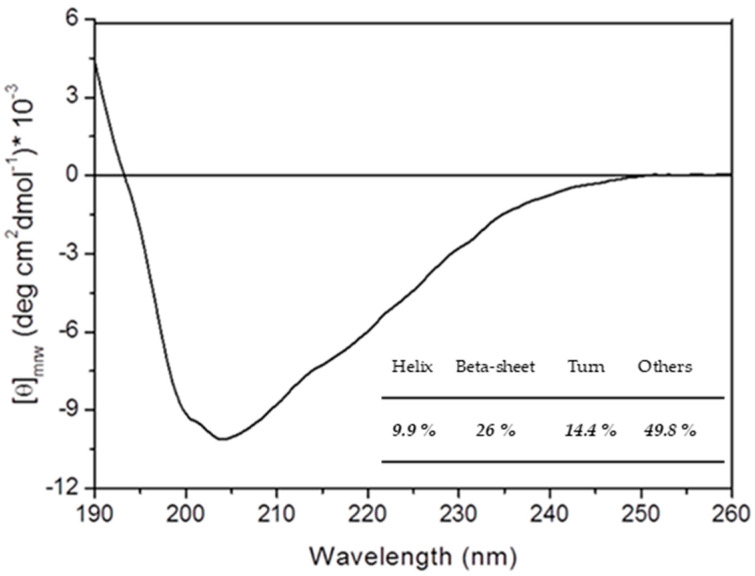
Far-UV CD spectrum of rPON2 (0.1 mg/mL) in a 0.1 cm quartz cuvette. Data are buffer subtracted, normalized for protein concentration, and reported as mean residue ellipticity. In the inset, the secondary structure content estimation is reported, which was performed with the webserver BestSel v1.3.230210.

A Circular Dichroism (CD) spectrum in the far ultraviolet region (Far-UV) of the monomeric protein was registered (Figure 4). Secondary structure content (Figure 4) obtained from spectra deconvolution using the webserver BestSel [28] confirmed that rPON2 monomer has a native folding [11,20].

### 2.2. Protein Stabilization

The monomeric form of rPON2 was incubated with several common additives routinely employed in recombinant protein production, such as trehalose and other osmolytes [29], that are reported to decrease protein aggregation [30,31] either by preferential hydration [32,33,34,35] or by acting as an amphiphilic interface between the protein hydrophobic surface and the polar solvent [36,37,38,39]

**Figure 5:**
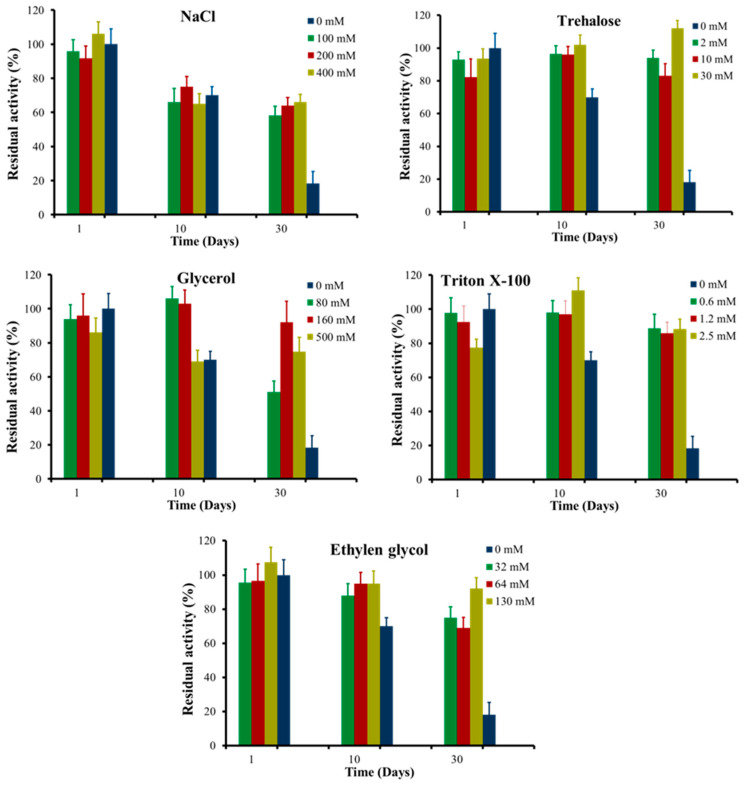
rPON2 residual activity over time (days), expressed as a percentage of the initial activity at day 0. Hydrolysis of pNP-C3 was monitored at 405 nm in 96-well plates, by using a multiplate reader. The chemical additives used were NaCl; trehalose; glycerol; Triton X-100; and ethylene glycol.

The protein esterase activity on 4-nitrophenyl propionate (pNP-C3) was measured at different time points during the incubation period. The results are expressed as the percentage of residual activity compared to the initial activity, which is set as 100%, and are reported in Figure 5.

rPON2 showed an initial specific activity of 0.62 U/mg for pNP-C3. Over the course of 1 month, the residual activity gradually decreased, being only 20% at the end point. The loss of activity could be due to the monomeric protein converting into the multimeric, less active form. It is worth noting that the stability of rPON2 obtained with this procedure is slightly higher than what was previously reported. Incubation of rPON2 with all the tested additives led to a significant increase in its stability in solution. Trehalose was the best additive, since no decrease in protein activity was observed at a concentration as little as 30 mM, followed by Triton X-100, for which the residual activity was around 90% independent of concentration. Ethylene glycol showed a concentration-dependent increase of residual activity from 70 to 90%, whereas glycerol and NaCl were the least effective in preserving protein activity, with around 70% and 60% residual activity, respectively. Moreover, at a higher concentration of glycerol, the protein residual activity was reduced, compared to lower concentrations. High concentrations of viscosity-generating solutes can affect the enzyme’s Michaelis–Menten constant [40,41,42], therefore affecting the catalytic efficiency [42,43].

**Figure 6:**
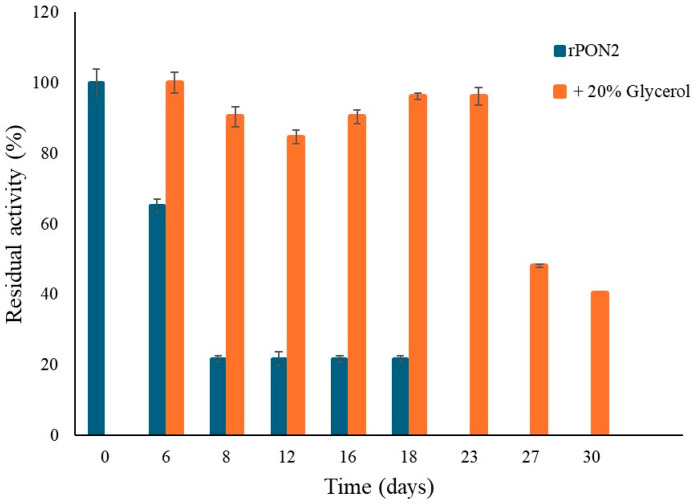
rPON2 residual activity over time (days), expressed as a percentage of the initial activity at day 0. Lactonase activity on 3oxoC12-HSL for the protein stored at 4 °C in the buffer only (blue) and at −20 °C in buffer with the addition of 20% glycerol (orange).

However, the effect of glycerol was different when samples of rPON2 were stored in 20% glycerol at −20 °C. Quite surprisingly, no loss of lactonase activity was detected when tested with the substrate 3oxoC12-HSL, until 23 days incubation, whereas at 27 and 30 days, a more significant loss of activity was observed (Figure 6). However, this represents a major improvement with respect to previous storage conditions.

### 2.3. Enzyme Kinetics

**Figure 7:**
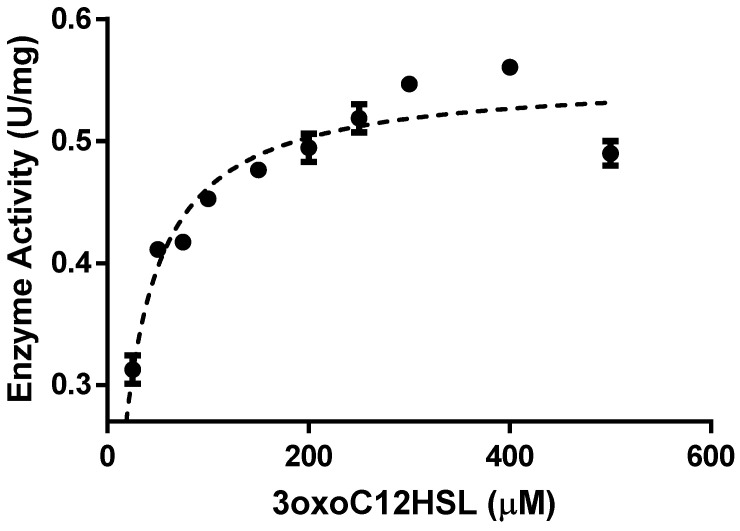
Michaelis–Menten curve of rPON2 for 3oxoC12-HSL at 37 °C (25–500 µM).

To verify if the new folding procedure had an impact on the high catalytic activity of the enzyme, purified rPON2 was used to perform kinetic measurements against 3oxoC12-HSL. In Figure 7, the Michaelis–Menten plot for the enzyme with 3oxoC12-HSL as the substrate is reported.

The kinetic parameters were derived from the Lineweaver–Burk linearization of the Michaelis–Menten plot. The Vmax was 0.55 (±0.01) µmol min^−1^ mg^−1^, the *k_cat_* was 0.33 (±0.01), s^−1^, the K_M_ was 21 (±3) µM, and the *k_cat_*/K_M_ was 1.6 (±0.2) × 10^4^ s^−1^ M^−1^.

### 2.4. Anti-Biofilm Assay

**Figure 8:**
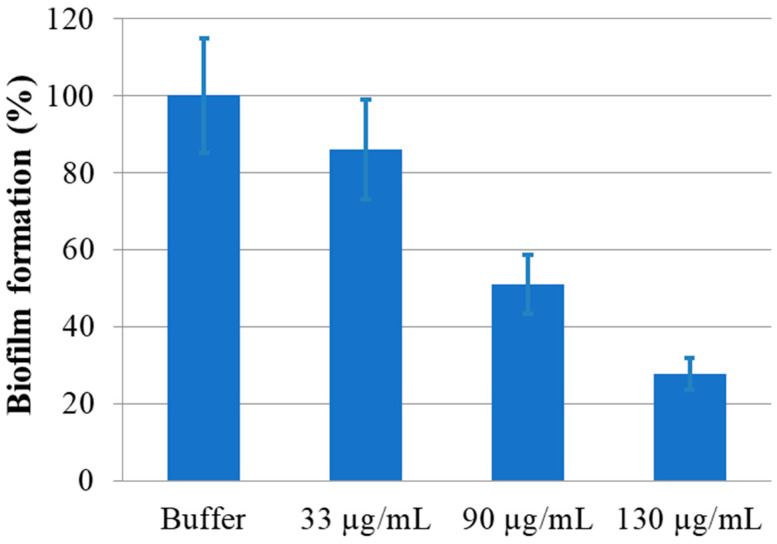
Biofilm inhibition capacity of rPON2, at the reported concentrations. The biofilm formation is expressed as a percentage of the biofilm formation compared to the control *PAO1* cells with protein buffer, quantified by the crystal violet assay.

The isolated protein monomer was also utilized in an in vitro anti-biofilm assay. Increasing concentrations of enzyme were incubated with a fixed amount of *Pseudomonas aeruginosa* (*PAO1*) cells overnight at 37 °C. The amount of biofilm formed was determined by using crystal violet, as described in the Section 4 and compared with the amount of biofilm formed in the absence of the protein, as a positive control [44]. The result of this assay is reported in Figure 8.

rPON2 was able to reduce the PAO1 biofilm formation by 50% at a concentration of 0.09 mg/mL. This reduction is increased to 70% when the protein is increased to 0.13 mg/mL, showing a good linear correlation between biofilm reduction and protein concentration. The biofilm reduction values reported here are even better than those reported in Mandrich et al. 2015 [20], showing that the protein obtained with this procedure is more effective in counteracting *PAO1* biofilm production.

### 2.5. Proteomic Approach

A comprehensive approach of direct molecular fishing (DMF) (see Section 4 for details) was performed utilizing purified rPON2 to capture potential interactors from HeLa cell extracts that were identified by mass spectrometry. The results from two independent experiments (A and B), employing different mass spectrometers with distinct resolution capabilities, are presented in Table 1, demonstrating common hits across both experiments. These findings underscore the reproducibility and reliability of the DMF approach in uncovering the putative interactors of rPON2.

**Figure 9:**
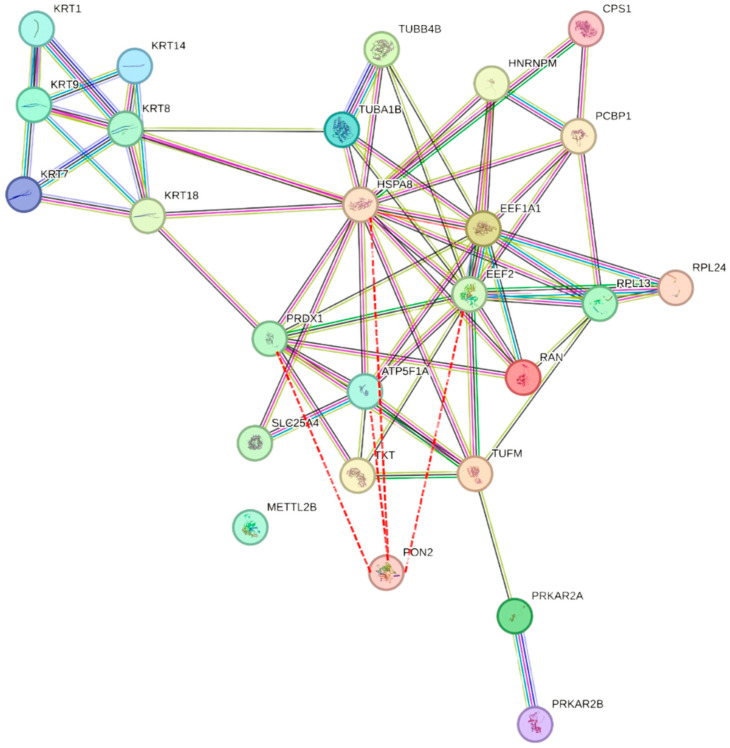
PON2 interactome. Interactions were predicted based on the String database (version 12.0). Known interactions (from curated databases and experimentally determined) are marked by pink and light blue lines and predicted interactions (gene neighborhood, gene fusion, and gene co-occurrence) are marked by green, red, and blue lines, respectively. The interactions that we identified commonly in multiple experiments with PON2 are shown by the red dotted lines.

Experiment A identified 423 interactors, while experiment B identified 155, with a note of caution regarding the latter, given the lower number of peptide hits identified (Table S1). Interactors from the BioGRID database [45] were also reported in Table S1. Among the identified interactors, 26 proteins were common in both A and B experiments, with only 6 of them reported in the BIOGRID Database. Figure 9 illustrates the PON2 interactome based on the data from Table 1 using the String program, providing a visual representation of the protein–protein interactions elucidated through DMF. Remarkably, out of these 26 proteins, 10 were also identified as PON2 interactors in a separate study by Teiber et al. 2014 [46], using co-immunoprecipitation, thus confirming the interaction of PON2 with these proteins through independent experiments. This dual confirmation enhances confidence in the identified interactors and reinforces the significance of the interactions in the context of PON2-mediated cellular processes.

## 3. Discussion

In this work, we reported an improved purification procedure for recombinant human PON2, which is remarkably less time-consuming (3 days versus 5 days) but is still able to provide a similar yield (1–2 mg) of the final product compared to the previously reported procedure. Far-UV CD data indicate that the protein can assume its correct folding during the on-column renaturation procedure. Moreover, the purified protein is fully active, with a specific activity of 0.55 ± 0.08 U/mg for 3oxoC12-HSL and 0.62 ± 0.04 U/mg for pNP-C3.

By looking at the specificity, the improvement was twofold and that is not consistent with the separation of monomer and dimer that indicated a ratio of 5.5 by the protein area. This discrepancy could be attributed to the presence of imidazole that could have an activating effect on the enzyme and is not present in the final samples.

Although the specific activity was in line with previous data, quite surprisingly, the K_M_ value (around 25 micromolar) was instead dramatically lower compared to model substrates such as 5-thiobutyl butyrolactone (TBBL) and pNP-C3 [20].

The affinity toward 3oxoC12-HSL compared favorably with the low concentrations found during bacterial infections, such as in infected wounds or cystic fibrosis patients.

This result points to 3oxoC12-HSL as a physiological substrate for PON2.

Accordingly, rPON2 exerts its biofilm inhibition activity on *PAO1* cells, showing that it is endowed with all the characteristics of human PON2. The combination of high affinity toward QS signal and the ability of the enzyme to interfere with biofilm formation in vitro holds promise for the potential use of PON2 in biotechnological applications.

Incubation of rPON2 at 4 °C with all the tested additives led to a significant increase in its stability in solution. Both trehalose and Triton X-100 showed the greatest effect, and thus, they are the most promising for developing an enzymatic formulation that can maintain the protein activity for a longer time, that is necessary for its use in the treatment of persistent bacterial infections. Storing rPON2 in 20% glycerol at −20 °C also preserved the activity if compared with storage at 4 °C in the absence of any additive, although after 23 days the activity started to decrease.

Despite the promising results on protein stability over time, we were constrained to keep the protein concentration at no more than 0.5 mg/mL, due to quick aggregation and consequent loss of activity. This represents a huge limit for downstream applications that require highly concentrated protein, such as obtaining crystals to solve tridimensional structure that is not yet available.

**Figure 10:**
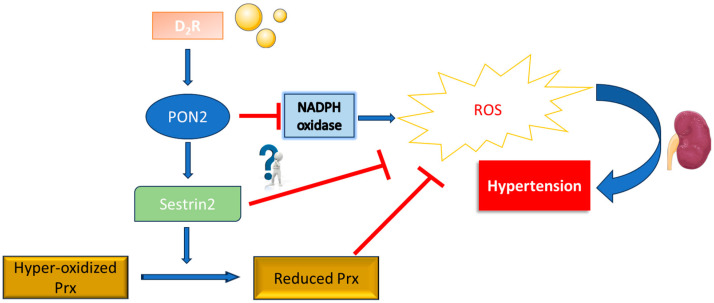
Sestrin 2 role in renal oxidative stress as modified from [47], and dopamine D2 receptor (D2R) upregulates Sestrin 2, reducing renal oxidative stress and maintaining normal blood pressure. D2R activation positively affects PON2, which, when active, inhibits NADPH oxidase and boosts Sestrin 2 expression. This dual action aids in reducing hyperoxidized peroxiredoxins (Prxs), effectively mitigating renal oxidative stress and ensuring normal blood pressure.

Thanks to the availability of the purified protein in its active form, we used the protein in the experiments of DMF. Although the difference between the two experiments A and B can be attributed to the instruments varying in resolution limits, unexpected is the low compliance of high-resolution mass spectrometry (HRMS) interaction list with the BioGRID data. One explanation could be that we used only cytosolic proteins; thus, we did not detect human somatin (STOM), a well-known PON2 interactor that binds membranes. Another important PON2 nuclear protein interactor KIAA1429, also known as Vir-Like M6A Methyltransferase-Associated (VIRMA) protein, seems to be missing. KIAA1429 interacts with Wilms’ Tumour 1-Associating Protein (WTAP) as well, representing a second link between PON2 and WTAP in addition to what we already described [11]. Noteworthily, in Table 1 and confirmed by Teiber data [46], there is a putative interaction of PON2 with Peroxyredoxyn 1. PON2 has been recently reported to be regulated by Sestrin 2, a well-known Peroxyredoxyn 1 interactor (Figure 10) [47].

To maintain cellular homeostasis, Sestrin 2 and its downstream molecules directly scavenge ROS or indirectly influence the expression patterns of key genes associated with redox, macroautophagy, mitophagy, ER stress, apoptosis, protein synthesis, and inflammation. Silencing PON2 increased Sestrin 2 ubiquitinylation (2.8-fold), decreased Sestrin 2 expression (30 ± 3%), and increased ROS production (1.3-fold), peroxiredoxin hyper-oxidation (2.9-fold), and lipid peroxidation (2.3-fold), while blocking the increase in Sestrin 2 that occurs with D_2_R stimulation [47]. Previously, the only known relationship of PDRX1 with PON2 was co-expression. The mechanisms by which PON2 performs this wide range of adjustments need to be further investigated.

By using the software Genemania (version 3.6.0) [48], it was possible to have an idea of the enrichment in each data set for genes participating in certain cell functions. By looking at the 26 common genes in experiments A and B, we detected enrichment with higher scores for nucleoid, mitochondrial matrix and membrane, response to glucagon, “de novo” posttranslational protein folding, chaperone-mediated protein folding, and “de novo” protein folding. For BioGRID data, instead, we had enrichment for the “insulin-like growth factor binding”, “sterol” and “cholesterol” transport, and cell–substrate junction assembly and organization. It is intriguing how from these data collectively an interplay emerges between insulin and glucagon pathways both involved in diabetes type 2, in which PON2 is involved [49,50,51].

## 4. Materials and Methods

### 4.1. Protein Expression, In Vitro Refolding, and Purification

rPON2 was purified using a modified version of the procedure reported in Mandrich et al. 2015 [20]. In brief, pT7-7 plasmid containing the *pon2* gene was transformed into *E. coli* BL21DE3 cells and streaked on LB agar plates containing ampicillin, as a positive selection. The colonies were picked, inoculated into seven liters of Luria–Bertani (LB) medium containing 100 μg/mL of ampicillin and grown overnight at 37 °C with vigorous bubbling of sterile air. The next day, after 3 h of IPTG induction (1 mM final concentration), cells were harvested by centrifugation (3000× *g*, 4 °C, 30 min) and stored at −20 °C until further use.

Around two grams of wet frozen cells were thawed and re-dissolved in 20 mL of lysis buffer (20 mM Hepes buffer pH 8.5; 0.5 mM CaCl_2_; 0.1 M NaCl). Cell disruption was achieved by sonication (Soniprep 150, MSE, Ltd., London, UK) using the following parameters: 40 cycles of 10 s “ON” and 30 s “OFF” and 15 µm amplitude.

A wash step was introduced to remove cell debris. Cellular pellet was resuspended 3 times in wash buffer (20 mM Hepes buffer pH 8.5; 0.5 mM CaCl_2_; 0.5% Triton X-100; 1 M urea), followed by a final wash with lysis buffer, to remove traces of Triton X-100. After each wash, the pellet was recovered by centrifugation (30,000× *g*, 4 °C, 20 min). The pellet obtained from the final centrifugation was re-suspended in 40 mL of Denaturing Buffer (20 mM Hepes buffer pH 8.5; 0.5 mM CaCl_2_; 5 mM glycine; 6 M Urea; 20 mM β-mercaptoethanol) and incubated at room temperature for 16 h to solubilize the inclusion bodies. The inclusion bodies were centrifuged to remove the insoluble material (13,000× *g*, 4 °C, 20 min), and 10 mL of nickel–NTA resin (New England Biolabs, Ipswich, MA, USA) was added to the supernatant. After 1 h of batch incubation at room temperature, the resin was loaded onto a column, and on-column renaturation was performed by decreasing the concentration of urea from 6 M to 0.25 M in a stepwise fashion. The resin was washed with 30 mL of each renaturing buffer (20 mM Hepes buffer pH 8.5; 0.5 mM CaCl_2_; 0.1% trehalose) containing a decreasing concentration of urea (4 M, 2 M, 1 M, 0.75 M, 0.5 M, 0.25 M), and the flow rate was maintained at 1 mL/min. After protein renaturation, the resin was washed with 25 mL of renaturing buffer containing 20 mM imidazole and eluted using 20 mL of renaturing buffer containing 200 mm imidazole. The sample was recovered in fractions of 2 mL, which were assayed for their lactonase activity. The fractions showing activity were pooled (about 12 mL total volume) and loaded onto a Hi-Load 26/600 Superdex 75 column (GE Healthcare Bio Sciences AB, Uppsala, Sweden) equilibrated with renaturing buffer. The protein was eluted with the same buffer at a flow rate of 1.5 mL/min. Gel filtration markers were run under the same conditions to obtain a calibration curve. Protein concentration of all the protein samples generated during the purification was determined with the Bradford assay, using a standard curve made with known amounts of bovine serum albumin [52].

### 4.2. Electrophoretic Analysis

An SDS-PAGE analysis was performed as described by Laemmli [53], at room temperature, using a 12.5% of acrylamide. Prestained Protein SHARPMASS VII (EuroClone, Milan, Italy) 6.5–270 kDa ladder was used as a molecular weight standard. The gel was stained using Coomassie Brilliant blue G250 (SERVA Electrophoresis GmbH; Heidelberg, Germany).

A Tris-glycine polyacrylamide gel system was used to analyze purified PON2 under non-denaturing conditions, as described by Davis [54], using 10% of acrylamide. The gels were run in a Mini-PROTEAN electrophoresis chamber (BioRad, Hercules, CA, USA) in running buffer (25 mM Tris, 192 mM glycine, pH 8.8), and the whole chamber was kept on ice to prevent heating of the sample. The proteins were then stained with a Silver Stain kit (BioRad, Hercules, CA, USA).

### 4.3. Circular Dichroism

The CD spectra of the protein were recorded under constant nitrogen flow on a Jasco J-815 spectropolarimeter (Jasco corporation, Tokyo, Japan), normalized for protein concentration and reported in terms of mean residue ellipticity (MRE), and represented by the symbol θ_MRW_ (measured in deg cm^2^ dmol^−1^) [55]. Buffer signal was subtracted from each protein spectra. A response time of 4 s, bandwidth of 2 nm, and scan rate of 20 nm/min were used. The protein concentration used was 0.1 mg/mL, and optical path length was 0.1 cm. The estimation of the amount of secondary structure of the protein was obtained through the deconvolution of spectra by means of the BestSel web analysis tool [28,56].

### 4.4. Enzymatic Activity Assays

Lactonase activity: Enzyme activity toward the substrate 3oxoC12-HSL was measured using a pH indicator, cresol red, to monitor the acidification of the medium induced by the hydrolysis of the lactone ring, as previously described by Chapman and Wong 2002 [57]. The time course of 3oxoC12-HSL hydrolysis was recorded on a Varian Cary 100 (Agilent, Santa Clara, CA, USA) equipped with a temperature controller set at 37 °C, and the wavelength was fixed at 572 nm, with an optical path length of 1 cm. The activity buffer contained 0.4 mM Hepes buffer pH 8.5, 150 mM NaCl, 0.12 mM cresol red (pKa 8.5 at 25 °C), and 5% Acetonitrile, the final concentration of substrate used was 0.5 mM. Before the assay, a calibration curve with acetic acid was performed, to obtain the rate factor (-OD/mole of H+) and calculate the enzyme specific activity. The activity (V_max_) was defined as µeq min^−1^ mg^−1^, which are the micromoles of free acid developed in one minute by 1 mg of r PON2.

Kinetic parameters were determined as follows: the reactions were performed at 0.4 mM Hepes buffer pH 8.5, 150 mM NaCl, 0.12 mM cresol red (pKa 8.5 at 25 °C), 5% Acetonitrile, and 1 µg of enzyme at 37 °C. After addition of the substrate to the mixture with the enzyme, the decrease in absorbance at 572 nm was monitored in 96-wells plates, using a reaction volume of 200 µL, in a Victor Nivo Multimode Microplate Reader (Pelkin Elmer; Waltham, MA, USA). Lactone substrate 3oxoC12-HSL was added from stocks of 5 and 50 mM in acetonitrile, and the percentage of acetonitrile was kept constant at 5% in all reactions regardless of the initial substrate concentration.

Kinetic parameters were measured with substrates ranging from 20 to 500 µM, measuring and subtracting the blank for each point. The initial velocity (v_o_) versus substrate concentration data were fitted to the Lineaweaver–Burk transformation of the Michaelis–Menten equation using the GraphPad (6.01 version) program.

Esterase activity: Enzyme activity toward the substrate pNP-C3 was analyzed by monitoring absorbance changes using a Victor Nivo Multimode Microplate Reader (Pelkin Elmer; Waltham, MA, USA) equipped with a 405/10 nm filter and set at 40 °C. Reactions were performed in 200 µL of activity buffer (20 mM Hepes pH 8.5; 0.5 mM CaCl_2_; 4% acetonitrile) using 96-wells plates. The final concentration of pNP-C3 was 2 mM. The spontaneous conversion rate of the substrate in the absence of the enzyme was measured independently and subtracted.

### 4.5. Inhibition of Biofilm Formation of Pseudomonas aeruginosa by rPON2

A stub of *Pseudomonas aeruginosa* (strain PAO1) was spread onto the solid medium Pseudomonas isolation agar (PIA) supplemented with Irgasan (100 mg/mL) and incubated overnight at 37 °C. Two colonies of *PAO1* were inoculated into 10 mL of Mueller Hinton broth (MHB) medium and grown at 37 °C, with 150 rpm shaking up to 0.2 O.D. 600 nm and diluted 1:1000, and then, the cells were inoculated in a 96-well plate (round bottom, polystyrene, TPP) in the presence or absence of rPON2 at different concentrations. *PAO1* (10^3^ CFU) was used as a control in the absence and presence of the enzyme buffer (20 mM Hepes buffer pH 8.5; 0.5 mM CaCl_2_, 0.1% trehalose; 0.25 M urea). After incubation at 37 °C for 24 h, the medium was removed, and the wells were washed three times with distilled water. The crystal violet staining was performed essentially as described by Marone et al. 2023 [58]; the absorbance of each well was read at 540 nm in a Victor Nivo Multimode Microplate Reader (PerkinElmer, Waltham, MA, USA).

### 4.6. Measurement of Enzyme Stability

Protein was diluted at 0.1 mg/mL in renaturing buffer (20 mM Hepes pH 8.5, 0.5 mM CaCl_2_, 0.1% trehalose, 0.25 M urea), supplemented with additives. Different concentrations of NaCl, trehalose; arginine; glycine; glycerol; triton X-100; and ethylene glycol were used. Solutions were stored at 4 °C, and an aliquot was withdrawn each day for a month and tested for its esterase activity. Protein was also stored at 0.05 mg/mL in 20% glycerol at −20 °C, and an aliquot was withdrawn every set number of days, as reported in Figure 6, and tested for its lactonase activity.

### 4.7. HeLa Cell Extract

A total of one million HeLa cells were seeded onto a plate and incubated with Dulbecco Modified Medium (Euroclone) with 10% fetal bovine serum (Euroclone) and 1% Penicillin–Streptomycin at 37 °C in a CO_2_ incubator to a confluence of 80%. The cells were lysed using modified radioimmunoprecipitation assay (RIPA) buffer (50 mM Tris-HCl pH 7.4, 150 mM NaCl, 1% NP-40, 1% Sodium Deoxy Cholate, 0.1% SDS) and incubated in ice for 30 min and centrifuged at 14,000× *g* for 10 min. The supernatant was collected and used for the DMF experiments.

### 4.8. Direct Molecular Fishing

Purified rPON2 (0.5 mg/mL) from size-exclusion chromatography was subjected to dialysis (Medicell International Ltd., London, UK) in 250 mL modified RIPA buffer, using 12–14 kDa membrane for 6 h at 4 °C. rPON2 was then incubated with 5 mL of HeLa extract (4.27 mg/mL) to aid the interactions for overnight at 4 °C on a see-saw rocker (Cole-Parmer Instrument Company, Vernon Hills, IL, USA). The extract was loaded and incubated for 1 h at 4 °C onto a column containing 5 mL of nickel–NTA resin (New England Biolabs, Ipswich, MA, USA). The affinity column was washed with RIPA buffer two times to discard the non-specific interactors. Proteins were eluted using 2 mL of Renaturing Buffer containing 300 mM imidazole.

### 4.9. Sample Preparation and Mass Spectrometry Analysis

The sample obtained from DMF technique was subjected to reduction using 5 mM Dithiothreitol (neoFroxx GmbH, Einhausen, Germany) in 8 M urea/50 mM NH_4_HCO_3_ and incubated for 1 h at 37 °C. The reduced sample was subjected to alkylation using 15 mM Iodoacetamide (Sigma-Aldrich, St. Louis, MO, USA) for 30 min in the dark at room temperature. The resultant sample was then diluted with three volumes of 50 mM NH_4_HCO_3_ to reduce the concentration of urea and digested by using 2 µL of Trypsin (Promega Italia Srl; Milan, Italy) at 37 °C, and after overnight incubation, 5% of formic acid (FA) was added to inhibit the digestion. Sample was then desalted using the ZipTipC_18_ (Merck Millipore Ltd.; Cork, Ireland) and analyzed by Linear Ion Trap Quadrupole LC-MS/MS QTRAP-4500 using the Analyst Software (version 1.6.2). Proteins were separated on LC column Acquity UPLC BEH C18 Column (130 A°, 1.7 µm, 2.1 × 50 mm) using Mobile Phase A, i.e., 0.1% FA in water/acetonitrile (98:2 *v*/*v*), and Mobile Phase B, i.e., 0.1% FA in acetonitrile/water (98:2; *v*/*v*). Instrument was operated in the Enhanced Mass Scan (EMS) and Enhanced Product Ion (EPI) modes, and samples were analyzed in the Information-Dependent Acquisition (IDA) mode, fragmenting the eight highest intense peaks of M1 scan and in the mass range of 300–1000 *m*/*z* with a threshold of 100 cps. The raw data were converted into mfg and mzML formats by using the ProteoWizard software 3.0.2, and the data were analyzed by using the Mascot and Trans Proteomic Pipeline (TPP) software (versions 2.8.0 and 6.1.0, respectively).

## Figures and Tables

**Table 1:** PON2 interactors confirmed from two independent experiments (A and B).

S. No	Gene ID	Protein Name
1	PON2	Paraoxonase2
2	CPS1	Carbamoyl-phosphate synthase [ammonia], mitochondrial
3	ATP5A1	ATP synthase F1 subunit alpha
4	HNRNPM	Heterogeneous nuclear ribonucleoprotein M
5	PRDX1	Peroxiredoxin1
6	KRT1	Keratin type II cytoskeletal 1
7	KRT7	Keratin type II cytoskeletal 7
8	KRT8	Keratin type II cytoskeletal 8
9	KRT9	Keratin type II cytoskeletal 9
10	KRT14	Keratin type II cytoskeletal 14
11	KRT18	Keratin type II cytoskeletal 18
12	RPL13	60S ribosomal protein L13
13	RPL24	60S ribosomal protein L24
14	EEF1A1	Elongation factor 1°1
15	EEF2	Elongation factor 2
16	EFTU	Tu translation elongation factor, mitochondrial
17	HSPA8	Hsp70-binding protein 1
18	RAN	GTP-binding nuclear protein Ran
19	TKT	Transketolase
20	PCBP1	Poly(rC)-binding protein 1
21	TUBA1B	Tubulin A1B
22	TUBB4B	Tubulin 4B
23	PRKAR2A	cAMP-dependent protein kinase type II alpha regulatory subunit
24	PRKAR2B	cAMP-dependent protein kinase type II beta regulatory subunit
25	METTL2B	tRNA N(3)-methylcytidine methyltransferase
26	SLC25A4	ADP/ATP translocase 1

## Data Availability

Data are available within the article or its Supplementary Materials.

## Supplementary Materials

The following supporting information can be downloaded at: https://www.mdpi.com/article/10.3390/molecules29112434/s1, Table S1: PON2 interactors.
