# Supplementary material for: The Human Paraoxonase 2: An Optimized Procedure for Refolding and Stabilization Facilitates Enzyme Analyses and a Proteomics Approach
Source: Molecules. 2024 May 22;29(11):2434. doi: 10.3390/molecules29112434 (PMC11173892; doi:10.3390/molecules29112434)
Supplement: Supplementary file 1 [file molecules-29-02434-s001.zip › molecules-3008342-supplementary.pdf]

Table S1. PON2 interactors from two experiments (A and B) and comparison with BioGRID data

**Exp.1**	**Exp.2**	**BioGRID**
AASDHPPT	ABCA1	A4GNT
ACAD10	ACOT4	ABCA2
ACM1L	ACOX2	ACPT
ACTA2	ACTC	ADAM30
ACTB	ADAP1	ADAMTS12
ACTBL2	SLC25A4	ADAMTS4
ACTBM	AKD1A	ADCK2
ACTG1	ANFC	ADRB2
ADH5	ANR39	AMIGO1
AGPAT5	ANR61	ANKRD46
AIMP1	APOB	ARSG
AIMP2	ARI5B	ARSK
AKR1A1	ARP2	ART5
AKR1B1	ATP5A1	ASIC4
AKR1B15	BMP8A	ATP2A2
AKR1E2	BRD9	**ATP6AP2***
AKR7A2	CB016	ATP6V1A
AKR7L	CC141	B3GNT2
ALAD	CCSE1	B3GNT3
ALB	CJ090	B3GNT7
ALDOA	CO2A1	B4GALT1
ANXA1	CO4A3	B4GALT3
ANXA2	CO4A6	BCAR1
ANXA2P2	COF1	BCKDHB
APEX1	CPSF6	BMP1
APOL2	CPSF7	C1QB
ARFGAP1	CPS1	C1QL4
ARG1	CX065	C9ORF78
ARMC6	DDX51	CA14
ARPC1B	DHX34	CACHD1
ATAD1	DYHC2	CACNA2D1
ATP10B	EEF1A1	CACNA2D2
ATP1A1	EEF2	CAMK2D
ATP1A2	EFNA4	CAMK2G
ATP1A3	EFTU	CANX
ATP1B3	FA53A	CARKD
ATP5A1	FAT4	CCDC47

ATP5B	FBRL	CCNF
ATP5J2	FBX41	CD1A
ATP6AP1	FLNA	CD1E
**ATP6AP2**	FNDC7	CDC6
ATP6V0D1	FXR1	CDHR4
ATXN10	GAGE10	CFC1
AZGP1	GTSC1	CHST10
BLVRA	HIRP3	CHST12
BP2	HME2	CHST14
BRF1	HMHA1	CHST7
BSG	HNRNPM	CHST8
BYSL	HNRNPU	CHSY1
C12ORF29	HNRPF	CLEC14A
C2ORF43	HSPA8	CLEC2B
C5ORF51	HXA10	CLEC2D
C9ORF64	HXK3	CMA1
CAB39	IF2P	CNNM1
CALCB	IGS10	CNTNAP3B
CAP1	IKZF4	COMT
CAPZA1	ITB4	CTSF
CAPZA2	ITIH4	CTSL
CCDC171	IYD1	CTSS
CCDC50	JADE1	CUL3
CCT2	JADE2	CYC1
CCT3	KRT18	DNAJC3
CCT4	KRT9	DNASE2B
CCT5	KRT7	DUSP9
CCT6A	KANK3	E
CCT8	PRKAR2A	E5
CD70	KCNC3	E5A
CDK7	KCNH5	E5B
CGNL1	KIF11	EDN3
CHORDC1	KIF1B	EGFR
CHRNA4	KIFC2	EMC1
CKMT1A	KPYM	ENPP1
CKMT1B	KRT1	ENTPD6
CKMT2	KRT14	ENV
CNTNAP1	KRT8	ERBB3
COPS5	LAMA5	EXT2
COPS6	**LAMB3**	EXTL3
CPA4	LMNA	FAM150B

CPOX	**LONP1**	FBXO6
CPS1	MAGB6	FLT3
CRKL	MAST2	FURIN
CRYZ	MBD2	GAL3ST2
CSNK2A2	MCM2	GALNT13
CSTA	MIDEAS	GP9
CTH	METTL2B	GPAT2
CTNND1	MRNIP	GPATCH8
CTSZ	MYT1L	GPR114
DCAF7	NLRP13	GRB2
DCD	NARF	GZMH
DCPS	NRAP	HK1
DDAH1	NT5D1	HLA-C
DFY3	O2AE1	HLA-DQA1
DKFZP68	OGFR	HLA-DQB1
DNAJA1	OR6S1	HLA-DRB1
DNAJA2	P4K2A	HLA-G
DNAJA3	PARI	HNRNPL
DPH5	PCBP1	HS2ST1
DPRHL2	PCM1	HS6ST2
DSC1	PK1L1	HSPA5
DSG1	**PLEKHA4**	HYAL2
DSP	PNT87	ICAM1
DST	POF1B	IMPAD1
DTWD2	PON2	INSR
DYNC2LI1	PRDX1	ISLR
ECDEC15A	PRKAR2B	ITGA4
EEF1A1	PRP16	ITGA6
EEF1A1P5	PTIP2	KCNK1
EEF1D	PX11C	KDELC1
EEF2	R13P3	KIAA1161
EFTU	RAN	KIAA1429
EIF2S1	RAP1B	KLK11
EIF3I	RGPD3	KLK15
EIF4A2	RIMS2	KLK5
ENO1	RL11	KLRAP1
ENO2	RL18	KLRB1
EPB41	RL18A	KLRC1
ERLIN1	RL26L	KLRC2
EXTL2	RL27A	KRAS
FAM118B	RL4	KTN1

FAM192A	RL8	**LAMB3**
FAM45	RN139	LINGO1
FAM50A	RPL13	LMLN
FAR1	RPL24	**LONP1**
FDPS	RS15A	LRFN3
FIGNL1	RS4Y2	LRIG1
FLG2	RSPO4	LRIG2
GALNT8	SC16B	LRP11
GAPDH	SCGR2	LRPPRC
GAPVD1	SCGR9	LRRC59
GGH	SEM6A	LRRTM1
GLUD1	SFPQ	LY6G5B
GNAI2	SNCAP	M
GNAI3	SPAG1	M6PR
GNAO	SPAG8	MAD2L2
GNAT3	SPB5	MAGEA10
GNPAT	TAF4B	MAP1LC3B
GPD1L	TAU	MARCH4
GPRC5A	TITIN	MOXD1
GRHPR	TKT	MPPE1
GTF3C1	TMC1	MR1
H0Y858	TRIM66	NCLN
HAUS6	TSH3	**NDUFA9**
HBB	TTLL8	NDUFS1
HDGF	TUBA1B	NDUFS3
HIP1R	TUBB4B	NDUFV1
HNRNPAB	TUBB5	NETO2
HNRNPD	UACA	NNT
HNRNPK	UBP11	NPC1
HNRNPM	UCP4	NR3C1
HNRPC	UT14C	NSP4
HRNR	VIME	NSP6
HSD17B4	YK032	NUP35
HSDL1	ZC11A	NUP98
HSP70BP1	ZEB1	NXPH4
HSP90AA1	ZN827	ORF7A
HSP90AB1		ORF7B
HSP90AB2P		ORF8
HSP90AB3P		ORF9B
HSP90AB4P		P2RY6
HSP90B1		P4HA1

HSPA8		PCDH20
HSPA9		PCNT
HSPD1		PGAP1
HTRA2		PIGG
ICK		**PLEKHA4**
IDH3A		PLXDC2
JUP		PNLIPRP1
KHSRP		POGLUT1
KMT2C		POMGNT2
KPNA2		POMK
KPRP		POMT2
KRT1		POR
KRT10		PPP2R5D
KRT12		PRELP
KRT13		PRG2
KRT14		PRSS53
KRT15		PTP4A2
KRT16		PTRF
KRT17		RAC1
KRT18		RAP1A
KRT19		RDH11
KRT2		RER1
KRT24		REV
KRT25		RHOA
KRT27		RLN1
KRT28		RNF128
KRT3		RNF130
KRT31		RNF167
KRT4		RNF170
KRT42		RUVBL2
KRT5		SAR1B
KRT6A		SCARB1
KRT6B		SCNN1A
KRT6C		SCNN1D
KRT7		SEMA4F
KRT71		SFTPC
KRT72		SH2D3C
KRT73		SIGLEC12
KRT74		SIL1
KRT75		SLC15A3
KRT76		SLC39A11

KRT77		SMOC1
KRT8		SPPL2B
KRT9		SPRTN
LANCL1		ST3GAL4
LARP7		ST3GAL5
LBR		ST3GAL6
LCLAT1		ST6GALNAC3
LDHA		ST8SIA4
LDHB		ST8SIA5
LGALS7		STOM
LMAN2		STRN3
LPCAT3		SULF2
LYZ		TAS1R3
MARC1		TAZ
MARC2		TCF3
MAT2A		TGFB1
MAT2B		TGOLN2
MCAM		TM2D3
MDH1		TMEM106A
MDH2		TMEM106B
METTL2B		TMEM219
MLST8		TMEM25
MROH8		TMEM30A
MRPL39		TMPRSS11B
MRPL43		TMPRSS13
MRPS35		TMPRSS2
MSH2		TMPRSS3
MTCH2		TMPRSS4
MTHFD2		TNFSF12-TNFSF13
MT-ND4		TNFSF13
MX1		TOR1AIP1
MYH6		TPP1
NACA		TRABD
NACAP1		TRAV20
NAGK		TRIM25
NAP1L1		TRIM67
NAP1L4		TUBG1
NCLN		TXNDC15
NDUFA10		UBR2
NDUFA13		UGT1A9

**NDUFA9**		UGT8
NFE2L2		UPK2
NGDN		UQCRC1
NLRP3		**UQCRC2**
NOVA1		UST
NPM1		UVSSA
NSDHL		VCP
NT5C3A		WNT1
NUP37		ZNRF4
NUP98		ZRANB1
OCIAD1		
OMA1		
OXA1L		
PCBP1		
PCBP2		
PCBP4		
PDCD7		
PDE4D		
PDHB		
PDHX		
PDIA6		
PDXK		
PEG10		
PEX3		
PIP		
POLDIP2		
PON2		
POTEF		
POTEI		
PPAP2C		
PPP1CA		
PPP1CB		
PPP1CC		
PPP2CA		
PPP2CB		
PRDX1		
PRDX2		
PRKAG1		
PRKAG2		
PRKAG3		
PRKAR2A		

PRKAR2B		
PRPS1		
PRPS1L1		
PRPSAP1		
PRPSAP2		
PRSS21		
PRSS3		
PRSS56		
PSIP1		
PSMD14		
PSMD7		
PTDSS1		
PTGR1		
PTPLAD1		
QPRT		
RAF1		
RAN		
RCN3		
RDH13		
RFC4		
RFC5		
RPL13		
RPL24		
RPL7		
RPLP0		
RPLP0P6		
RPS2		
RPS9		
RPSA		
S100A8		
S100A9		
SCAMP3		
SCD		
SCGB1D2		
SEC13		
SEC61A1		
SEC61A2		
SEH1L		
SEPT2		
SERBP1		
SERPINB12		

SERPINB3		
SERPINB6		
SERPINB8		
SERPINH1		
SF3B2		
SF3B4		
SFXN3		
SFXN4		
SGTA		
SHCBP1		
SHROOM3		
SKP1		
SLC16A3		
SLC22A18		
SLC25A11		
SLC25A12		
SLC25A13		
SLC25A3		
SLC25A31		
SLC25A32		
SLC25A4		
SLC25A40		
SLC25A5		
SLC25A6		
SLC30A7		
SLC35B2		
SLC7A5		
SLC7A6		
SMAD4		
SOAT1		
SOD1		
SOGA2		
SORD		
SRR		
SSR1		
STK32A		
STRAP		
SUCLG1		
SYPL1		
TALDO1		
TAMM41		

TBRG4		
TCEA1		
TCEA2		
TCP1		
TFB2M		
TFG		
TIM50		
TKT		
TMEM165		
TMEM33		
TMEM39B		
TOMM40		
TOR1AIP1		
TPM1		
TPM2		
TPM3		
TPM3L		
TPM4		
TRAP1		
TRIM4		
TRIP11		
TTC1		
TUBA1A		
TUBA1B		
TUBA3C		
TUBA3E		
TUBA4A		
TUBA8		
TUBAL3		
TUBB		
TUBB2A		
TUBB3		
TUBB4A		
TUBB4B		
TUBB6		
TUBB8		
TWF1		
TWF2		
TXNL1		
UBB		
UCHL5		

UFD1L		
UGT1		
UNG		
**UQCRC2**		
VDAC2		
VPS26A		
VTA1		
VTA1		
WARS2		
WBP2		
WDR5		
XRCC6		
ZAGL1		
ZC3HAV1L		
ZNF394		
ZNF418		
ZNF536		

*Proteins in common between Biogrid and Exp A or B are indicated in bold.
